# Knowledge, attitude and practices of snakebite management amongst health workers in Cameroon: Need for continuous training and capacity building

**DOI:** 10.1371/journal.pntd.0006716

**Published:** 2018-10-25

**Authors:** Fabien Taieb, Timothée Dub, Yoann Madec, Laura Tondeur, Jean Philippe Chippaux, Matthew Lebreton, Raphael Medang, Françoise Ngnedjou Nwabufo Foute, Désiré Tchoffo, Julien Potet, Gabriel Alcoba, Eric Comte, Ellen M. Einterz, Armand S. Nkwescheu

**Affiliations:** 1 Emerging Diseases epidemiology unit, Institut Pasteur, Paris, France; 2 Centre for Translational Science, Institut Pasteur, Paris, France; 3 « Mother and Child facing Tropical Infections » unit, Institut de Recherche pour le Développement, Paris, France; 4 Mosaic, Yaounde, Cameroon; 5 Metabiota, Yaounde, Cameroon; 6 IECD, Institut Européen de Coopération et de Développement, Paris, France; 7 Distant Production House University, Yaounde, Cameroon; 8 Cameroon Society of Epidemiology, Yaounde, Cameroon; 9 MSF Access Campaign, MSF, Paris, France; 10 Division of Tropical and Humanitarian Medicine, Geneva University Hospitals, Geneva, Switzerland; 11 Centre International de Recherches, d'Enseignements et de Soins (CIRES), Yaounde, Cameroon; 12 Center for Global Health, Indiana University, Indianapolis, IN, United States of America; College of Health Sciences, Bayero University Kano, NIGERIA

## Abstract

**Background:**

Snakebite has only recently been recognized as a neglected tropical disease by the WHO. Knowledge regarding snakebites and its care is poor both at the population level, and at the health care staff level. The goal of this study was to describe the level of knowledge and clinical practice regarding snakebite among health care staff from Cameroon.

**Methods:**

A two-day training dedicated to snakebite and its care was organized in 2015 in Yaoundé, capital city of Cameroon. A total of 98 health care staff from all over Cameroon attended the training. Prior to and after the training, an evaluation quantified the attendees’ level of knowledge. Pre- and post-training evaluations were compared to assess knowledge improvement.

**Results:**

Overall, prior to the training knowledge regarding snakebite and care was poor, and wrong beliefs that “pierre noire” or tourniquet were useful in case of snakebite were common. Knowledge was statistically improved after the training.

**Conclusion:**

Trainings dedicated to all type of health care staff towards snakebite to improve care are needed, this training must take into consideration the context and the targeted population.

## Author summary

In this manuscript, I report the general knowledge regarding snakebite envenomation and its care before and after a two-day course that was organized in Yaounde, the capital city of Cameroon, in late 2015. Snakebite is a public health issue, particularly in sub-Saharan Africa, where access to antivenoms is dramatically insufficient. Anticipating the decision of the World Health Organization that added snakebite envenomations to the list of neglected tropical diseases in June 2017, Cameroon has joined the African Society of Venimology and several Non-Governmental Organizations, including Médecins sans Frontières, to put in place a national snakebite envenomation control strategy. The article describes the state of knowledge of health care workers who participated in the training about snakebite envenomations in Cameroon at the time of the launching of the National Snakebite Envenomation Control Program. It also describes the improvement of knowledge thanks to the training. In conclusion, the results obtained highlight the need for continuous training of the health care staff and capacity building over the next few years.

## Introduction

Worldwide, the burden of envenomation due to snakebites is imprecisely estimated. Mathematical models estimate of the global number of snakebite envenomation cases range from 420,000 to 2,682,500 annually [1, 2]. In sub-Saharan Africa, the number of persons treated in health centers for snakebite envenomation is estimated at 315,000 cases per year with more than 9,000 amputations and 7,000 deaths, although these numbers are probably greatly underestimated [3]. This recently led the World Health Organization (WHO) to declare snakebite envenomation a neglected tropical disease [4] opening access to new strategies and resources [5].

In Cameroon, a recent article highlighted the weakness of national surveillance systems leading to an under-estimation of the actual number of snakebites [6]. In 2015 after 22 weeks of surveillance, the national surveillance system of Cameroon reported 848 cases of snakebites and 10 associated deaths [7]. In the northern part of the country where venomous snake density is high [8] several studies have already been conducted on this subject, although these now date from over a decade ago. In 2002, a study estimated the annual incidence of envenomation to be 200 per 100,000 inhabitants. Another study reported that 85% of reported snakebites were due to *Echis ocellatus with* lethality as high as 23.9% [9].

In rural regions, where most snakebites occur [3], the general population’s awareness of appropriate behavior in response to snakebite is poor. A study in Nigeria showed that most patients, prior to presentation at hospital, had attempted at least one potentially deleterious first aid measure [10]. While the population as a whole needs to be educated, the training of health care staff and the standardization of care should not be neglected either [10]. In Ghana, an intervention oriented towards improving snakebite medical care ability and adherence to protocols led to a significant improvement in care and significant decrease in snakebite mortality when pre- and post-training outcomes were compared [11].

The goal of the present study was to describe the level of knowledge and clinical practice regarding snakebite among health care staff from Cameroon before and after a training on herpetology, snakebite epidemiology, clinical care of patients and antivenoms.

## Methods

In November 2015, in order to assess and improve Cameroonian health care staff knowledge on snakebites and standard of care of snakebite cases, 98 health professionals from around the country were invited by the Cameroon Ministry of Health (MoH), to attend a two-day training in Yaoundé (Cameroon). The participation to this training was based on a voluntary approach. Criteria for training participation was to be Medical Doctor, nurse, pharmacist, dental surgeon or biologist working in a health facility. The training and its content was announced in media (radio and press), and posters were pasted down to the level of health facilities (public and private). Under the authority of the MoH, this training was organized by the Cameroon Society of Epidemiology (CaSE) in collaboration with the Centre International de Recherche, Enseignement et Soins (CIRES, Cameroon), the WHO, Institut Pasteur (France), Médecins Sans Frontières—Doctors without Borders (MSF), Geneva University Hospitals (Switzerland), Société Africaine de Venimologie–African Society of Venimology (SAV–ASV) and the Indiana University Center for Global Health (USA).

The training consisted of sessions devoted to the identification of snakes, snakebite epidemiology, pathophysiology, terminology, semiology, and clinical care and use of antivenoms (e.g. rationale for administration, surveillance, side effects, composition and properties). The training was part lecture and part practical exercises in small groups.

In order to assess baseline knowledge on snakebites and associated care and to assess how the training could improve this knowledge, an evaluation was submitted to attendees, both before and after the training. This evaluation consisted of the same 38 multiple choice questions covering all topics of the training. The evaluations were not corrected with participants during the two days of training. So, the correct answers were not disclosed to the participants after the first evaluation, but only at the end of the training and after the final evaluation was conducted in order to minimize a potential test effect bias. Individual scores were then calculated by attributing 1 point if the answer to the question was correct (i.e. all correct items identified in case of multiple correct answers) and 0 point otherwise (see supplementary information). The score therefore could range from 0 (worst) to 38 (best). Three additional questions specifically dealing with antivenom characteristics were added to the post-evaluation questionnaire although not considered in the scoring. Evaluation tests were anonymous, but an identification number randomly allocated to each attendee was used to link the pre- and post-training evaluations. In the pre-training questionnaire, the following information was also recorded: gender, age, profession, region of work, type of health care facility, and public or private sector.

Data from attendees’ questionnaires were entered into an electronic database using the Wepi tool (EpiConcept, France).

Characteristics of the attendees were compared between groups using the chi-2 or fisher test depending on the sample size for the categorical variables, and using the Wilcoxon non-parametric test for continuous variables. The score was compared between groups using the Wilcoxon non parametric test when two-groups were considered and the Kruskall-Wallis non parametric test otherwise. Pre- and post-training scores were compared using the Wilcoxon test for paired data. P-values <0.05 were considered significant. All statistical analyses were conducted using Stata 13 (Stata Corp., College Station, TX, USA).

## Results

### Characteristics of the attendees

Of the 98 health professionals who attended the training, 17 (17.3%) completed only the pre-training evaluation test, 19 (19.4%) completed only the post-training evaluation test, and 62 (63.3%) completed both pre- and post-training evaluations (Table 1). All the 98 attendees completed at least one of the evaluations. The majority of attendees came from Centre region (40.8%), the remaining were from all over the countries (except one participant from Chad) (Table 1).

**Table 1:** Characteristics of the attendees.

	All	Only pre- or post- training Evaluation	Pre- and post-training evaluations	P-value
	n = 98	n = 36	n = 62	
**Gender**				0.44
**Male**	57 (58.2)	22 (61.1)	35 (56.5)	
**Female**	38 (38.8)	12 (33.3)	26 (41.9)	
**Not specified**	3 (3.0)	2 (5.6)	1 (1.6)	
**Age (in years)**				0.71
**n**	92 (93.9%)	35 (97.2%)	57 (91.9)	
**median (IQR)**	37 (31–45)	36 (29–44)	36 (31–46)	
**Occupation**				<0.001
**Medical doctor**	36 (36.7)	8 (22.2)	28 (45.2)	(0.17*)
**Nurse**	23 (23.5)	2 (5.6)	21 (33.8)	
**Pharmacist**	10 (10.2)	3 (8.3)	7 (11.3)	
**Other**	10 (10.2)	4 (11.1)	7 (11.3)	
**Not specified**	19 (19.4)	19 (52.8)	-	
**Sector**				<0.001
**Public**	46 (60)	6 (16.7)	40 (64.5)	(0.03*)
**Private**	31 (40)	11 (30.6)	20 (32.3)	
**Not specified**		19 (52.8)	2 (3.2)	
**Region**				<0.001
**Center**	40 (40.8)	8 (22.2)	32 (51.6)	(0.69*)
**North**	11 (11.2)	3 (8.3)	8 (12.9)
**West**	15 (15.3)	2 (5.6)	13 (21.0)
**East and South**	7 (7.2)	2 (5.6)	5 (8.1)
**Other/unspecified**	25 (25.5)	21 (58.3)	4 (6.4)
**Care of snakebite the previous year**				<0.001
**No**	45 (45.9)	9 (25.0)	36 (58.1)	(0.78*)
**Yes**	33 (33.7)	8 (22.2)	25 (40.3)	
**Unspecified**	20 (20.4)	19 (52.8)	1 (1.6)	
**Previously attended a training**				<0.001
**No**	67 (68.4)	14 (38.9)	53 (85.5)	(0.68*)
**Yes**	10 (10.2)	3 (8.3)	7 (11.3)	
**Unspecified**	21 (21.4)	19 (51.8)	2 (3.2)	

Data are n (%) unless specified

IQR: inter quartile range

* when the “unspecified” category is not considered

Most attendees were medical doctors (n = 36, 36.7%) or nurses (n = 23, 23.5%). The remaining attendees were pharmacists (n = 10, 10.2%), or had other occupations (n = 10, 10.2%) including laboratory staff and students; the remaining 19 (19.4%) did not specify their occupation. Attendees were based in several different regions of Cameroon, although a large proportion were from the Center Region (n = 40, 40.8%). Only a minority of attendees (n = 10, 10.2%) had previously attended a training dedicated to snakebite management. Of the 59 medical doctors and nurses, 9 (15.2%) declared having been previously trained. One third (n = 33, 33.7%) of the attendees reported having taken care of patients with snakebite in the previous year. This proportion reached 50.9% when only the medical doctors and nurses were considered. Five of them declared having dealt with more than 10 cases of snakebites in 2015.

Overall, evaluations of attendees who completed both pre- and post-training evaluations were not different from those who participated in only pre- or post-training evaluations.

### Evaluation of overall knowledge

Before the training, overall knowledge on snakebites estimated from 79 attendees was poor, with a median (inter quartile range [IQR]) score of 10.0 [8.0–15.0]. This score was low whatever the occupation of the attendee; the median [IQR] score was 12.0 [8.5–18.0], 9.0 [7.0–13.0] and 10.0 [6.0–12.0] in medical doctors, nurses, and other staff, respectively (p = 0.045) (Table 2). The pre-training score was significantly higher in those who had previously been trained (p = 0.011), but did not differ between those with and without previous experience of snakebite care (p = 0.66).

**Table 2:** Attendees score by occupation.

	Medical doctors	Nurses	Other	p-value
Pre-training (n = 79)*	12.0 (8.5–18.0)	9.0 (7.0–13.0)	10.0 (6.0–12.0)	<0.05
Post-training (n = 81)**	28.0 (25.5–30.0)	22.0 (21.0–26.0)	24.0 (23.0–26.0)	< 0.001
Gain (n = 62)***	15.0 (11.5–18.0)	11.0 (10.0–15.0)	16.0 (13.0–17.0)	<0.05

*Pre-training score was calculated on 36 medical doctors, 23 nurses and 20 other professionals.

**Post-training score was calculated on 28 medical doctors, 21 nurses and 32 unspecified or other professionals.***Score gain was calculated on 28 medical doctors, 21 nurses and 13 other professionals.

After the training, the overall knowledge on snakebites of the 81 attendees was higher than prior to the training. The median [IQR] score was 26.0 [22.0–28.0]. The median [IQR] score post-training was higher in medical doctors than in nurses and other health professionals (28.0 [25.5–30.0], 22.0 [21.0–26.0] and 25.0 [23.0–26.0], respectively; p<0.001) (Table 2). The post-training score was also significantly higher in those who had previously attended a training (p = 0.041), but again the post-training score did not differ between those with and without previous experience of snakebite care (p = 0.84).

In the 62 participants who participated to both evaluations, as compared to pre-training, the post-training overall score was significantly increased (p<0.001) whatever the occupation (Table 2), indicating an improvement in knowledge for all individuals during the training.

### Evaluation of first-aid and care knowledge

In the evaluation, 26 questions were related to first-aid and care of snakebite victims. The sub-score related to care was low, and no significant difference in the scores related to this topic was detected between the different occupations of participants (p = 0.07). The training resulted in significantly improved knowledge regarding first-aid and care knowledge (p<0.001) (Table 3). In more details, prior to the training, only 36% of attendees correctly responded that the “*pierre noire*” (a carbonized cow bone fragment) was not useful when dealing with snakebite, and the proportion was similar regardless of attendees’ occupation (p = 0.40). This proportion increased to 96% after the training. Similarly, only 63% correctly declared pre-training that using a tourniquet above the snakebite was not recommended, and this result did not differ by occupation (p = 0.20). Post-training, 100% of participants declared that it was not recommended.

**Table 3:** Attendees score by occupation specific to epidemiology and medical care.

	Medical doctors	Nurses	Other	p-value
Pre-training (n = 79)* Epidemiology-ecology **Clinical care**	4.0 (3.0–5.0) 7.5 (6.0–14.0)	3.0 (1.0–4.0) 7.0 (5.0–10.0)	3.0 (2.0–4.5) 6.0 (5.0–8.5)	0.07 0.07
Post-training (n = 81)** Epidemiology-ecology Clinical care	8.0 (6.5–9.0) 20 (18.0–21.5)	6.0 (5.0–7.0) 17.0 (15.0–18.0)	7.0 (6.0–8.5) 17.0 (15.0–20.5)	0.002 0.003
Gain (n = 62)*** Epidemiology-ecology Clinical care	4.0 (3.0–5.5) 11.0 (7.0–13.5)	3.0 (2.0–4.0) 8.0 (7.0–12.0)	5.0 (4.0–5.0) 11.0 (9.0–12.0)	0.04 0.25

Regarding care, prior to the training 63% reported correctly that not all snakebites require administration of antivenom. This proportion was independent of attendees’ occupation (p = 0.23). After the training, this proportion reached 95%. Pre-training, only 52% reported that antivenom administration was intravenous versus intramuscular or orally (p = 0.60 across occupations), and it was still only 75% post-training (p = 0.09 across occupation).

### Evaluation of ecology and epidemiology knowledge

Twelve questions were related to the ecology of snakes and to the epidemiology of snakebites. Overall, the sub-score on epidemiology and ecology was low prior to the training and did not differ by occupation (p = 0.07) (Table 3). The training resulted in significantly improved knowledge regarding epidemiology and ecology (p<0.001).

### Attendee satisfaction of the training

Of 78 attendees who rated the training, 77 (98.7%) were satisfied and the median (IQR) score they gave to the training was 17/20 (15–18). They also stated that such training would be useful if repeated either annually (n = 37, 46%) or every two years (n = 37, 46%).

## Discussion

Antivenoms are available in Africa, but two key issues need to be addressed. First, access to care for victims of snakebites must be improved through community awareness and education [12, 13]. Second, clinical practice in cases of snakebite should be improved. The Cameroon Ministry of Public Health and the Cameroon Society of Epidemiology (CaSE), wishing to support the global fight against snakebite envenoming, co-organized this training in collaboration with national and international partners.

This training addressed the need expressed by the MoH to improve knowledge regarding snakebite management and to encourage good clinical practices among health care staff who are in direct contact with patients bitten by snakes. This was the opportunity to make health authorities and health care staff more aware of the burden of snakebites and was to build medical awareness and skills that might foster the medium-term implementation of epidemiological and clinical research programs aimed at improving quality of care and knowledge coming from the field. This last point is of particular importance, since the lack of data collection and standardization of care impedes the drawing of conclusions about the efficacy of different practices [14]. However, such trainings concern the whole medical personnel, since in Africa whatever the occupation, all of them are involved in snakebite management, including biologists (at least one case among the participants in the training) and midwives who are used to treat severe hemorrhages occurring in pregnant women.

Evaluation of first-aid and care knowledge showed that the level of knowledge was very low before this training as in most professions worldwide, where snakebite is neither an undergraduate nor a post-graduate medical or nursing topic. In Africa, as in many parts of the world, the teaching of management of bites or stings due to venomous animals is no longer compulsory—or is very limited—in health/medical training facilities with some rare exceptions [15–17].

General knowledge of attendees improved significantly after the training. It is interesting to note that misconceptions concerning traditional practices such as incisions and the use of “*pierre noire*” or tourniquets were similar to that of the general population prior to the training [18, 19]. While emphasizing good clinical practices, the training also underscored the potential adverse health impact of these kinds of practices.

Several studies showed that a large proportion of snakebites are exclusively treated by traditional healers [18, 20–22]. Because in many cases snakebite is associated with notions of magical phenomena or bad omens, patients may be inclined to seek help from a traditional practitioner before visiting a hospital and contemplating the purchase of costly antivenom. As a result, traditional practitioners may often be in earlier contact with patients. With a better understanding among both traditional and modern practitioners, traditional healers could be key to a timelier referral of patients to health care facilities that are able to administer antivenoms. To advance awareness and determine the best place of traditional medicine in the cascade of care, workshops with traditional practitioners might be wisely included in future trainings.

We achieved our main goal of accessing health care staff from around the country who are in direct contact with snakebites, although health care staffs from the Centre Region were over-represented. The reason for this was simply geographic, as the training took place in that region. It shows the importance of decentralizing such trainings in order to access the country’s health care staff more broadly. According to the Ministry of Public Health, the geographical distribution of attendees was consistent with the distribution of reported snakebite cases in the country, apart from the Far North region, the second region in the country below the Centre region in terms of snakebite occurrence, due to security issues making travel and absenteeism of health professionals complicated.

Interestingly, only a minority of attendees had previously attended a training dedicated to snakebite care despite being frequently confronted by these situations. Given the high initial level of misconception observed among attendees, it is important to offer trainings regarding care of snakebites to the widest audience possible. This evaluation also showed a lower level of knowledge for nurses compared to medical doctors, making nurses an essential target for these trainings. This is supported by the fact that in remote settings and in a context of limited medical human resources, a lot of medical care is provided by nurses.

Training attendees showed a high level of satisfaction with the training, and reported that such trainings should be organized regularly. However, in this evaluation, the pre-training score was found to be low even among those who had already attended a training on snakebites. The score among those attendees was not different from the score of attendees who had never been trained on the topic. However, the content, quality, duration and timing of the previous trainings are unclear and the post training score was significantly better even among those who had previously been trained, showing the positive impact of regular high quality training.

Our evaluation presented some limitations. The design with a pre- and post- test evaluation could have affected our results due to the test effect. Indeed improvements in the scores can resulted from the test itself, attributable to factors such as participants’ remembering questions or questions raising awareness and triggering learning after the pre-test, independently of the subsequent intervention. Still, correct answers were not disclosed after the first evaluation, but only at the end of the training and after the final evaluation was conducted. We organized a final restitution of the results in order to highlight some weaknesses in initial knowledge or beliefs, major areas for improvement, and knowledge that was not yet consolidated. Another limitation was the lack of long-term evaluation of the knowledge retention. This was a time and space limited exercise and such experiments should be multiplied to standardize training and compare the results obtained.

We aimed at reporting an experience in order to improve our training as well as other actors' training on the same topic. Attendees appreciated the training, especially alternation between theoretical knowledge and practical workshops as well as the duration of the training, which was compatible with their workload. Attendees appreciated also the transversal approach of the training. They asked for a strengthening of training in term of health care management, antivenoms, and involvement of traditional practitioners and midwives in future trainings.

## Conclusion

Surveillance of snakebites is essential to better understand its epidemiology. Reporting of cases must be improved to provide accurate data and to facilitate stock management, training implementation, and appropriate interventions. Now that snakebites are recognized as a Neglected Tropical Disease, a global, adapted and sustainable approach is required. Training of health care personnel represents a key aspect of this global approach.

The evaluation of this training showed there is a need to encourage academic teaching regarding the management of venomous bites and stings in all health institutions in sub-Saharan Africa. This does not preclude the development of continuing education on this topic involving all health personnel. We recommend that continuing education be provided in two ways: first, the training of trainers at the national level that can benefit from international support, and health personnel training at the local level under the supervision of national health authorities.

The training was organized under the patronage of the Ministry of Health, with the support of national and regional institutions. This vertical approach for organization, as recommended by international bodies such as WHO, is both simpler and faster to achieve. A horizontal approach for organization based on a regional organization could ensure a better local representativity and better consideration of cultural diversity of the attendees, but could also generate some difficulties in term of homogeneity and accuracy of the content of the training. Regarding the latter, we believe that a transversal and multidisciplinary approach is necessary. The content of the training should be based on multidisciplinary international, national and local expertise taking in account local beliefs and practices, as far as they are appropriate.

This evaluation showed a significant difference in knowledge acquisition according to personnel occupation. Attention should be on a) first aid to avoid unnecessary and dangerous deeds, sometimes applied in health centers, b) treatment with appropriate antivenoms, and c) the importance of associating symptomatic treatments with antivenom immunotherapy in order to reduce treatment failure and avoid complications. Community based efforts should be increased to promote prevention, first-aid and to improve health-seeking behaviors.
